# The Concentration of BTEX in the Air of Tehran: A Systematic Review-Meta Analysis and Risk Assessment

**DOI:** 10.3390/ijerph15091837

**Published:** 2018-08-24

**Authors:** Mehrnoosh Abtahi, Yadolah Fakhri, Gea Oliveri Conti, Margherita Ferrante, Mahmoud Taghavi, Javad Tavakoli, Ali Heshmati, Hassan Keramati, Bigard Moradi, Nazak Amanidaz, Amin Mousavi Khaneghah

**Affiliations:** 1 Department of Environmental Health Engineering, School of Public Health, Shahid Beheshti University of Medical Sciences, Tehran, Iran; Mehrabtahi@sbmu.ac.ir; 2 Department of Environmental Health Engineering, Student Research Committee, School of Public Health, Shahid Beheshti University of Medical Sciences, Tehran, Iran; 3 Department of Medical, Surgical Sciences and Advanced Technologies “G.F. Ingrassia”, University of Catania, 95131 Catania, Italy; olivericonti@unict.it; 4 Department of Environmental Health Engineering, School of Public Health, Social Development & Health Promotion Research Center, Gonabad University of Medical Sciences, Gonabad, Iran; taghavi66@yahoo.com; 5 Department of Food Science and Technology, Faculty of Agriculture, Jahrom University, Jahrom, Iran; ja_tavakoli@yahoo.com; 6 Nutrition Health Research Center, Hamadan University of Medical Sciences, Hamadan, Iran; ali_heshmaati@yahoo.com; 7 Department of Environmental Health Engineering, School of Public Health, Semnan University of Medical Sciences, Semnan, Iran; Hkramatee@gmail.com; 8 Research Center for Environmental Determinants of Health (RCEDH), Kermanshah University of Medical Sciences, Kermanshah, Iran; b.moradi@gmail.com; 9 Environmental Health Research Center, Golestan University of Medical Sciences, Golestan, Iran; Amanidaz_n@yahoo.com; 10 Department of Food Science, Faculty of Food Engineering, State University of Campinas (UNICAMP), 13083-862 Campinas, São Paulo, Brazil

**Keywords:** BTEX, systematic review, meta-analysis, health risk, Tehran, air pollution, benzene, toluene

## Abstract

In the current study, the concentration of some pollutants which are categorized as volatile organic compounds (VOCs), including benzene (B), toluene (T), ethylbenzene (E), and o-xylenes (o-X), in the air of Tehran was evaluated by the aid of a systematic review and meta-analysis approach. Also, the health risk for the exposed population was estimated using the recommended methods by the Environmental Protection Agency (EPA). The rank order based on their concentration in BTEX was benzene (149.18 µg/m^3^: 31%) > o-xylene (127.16 µg/m^3^: 27%) > ethylbenzene (110.15 µg/m^3^: 23%) > toluene (87.97 µg/m^3^: 19%). The ratio B/T in this study was calculated as 1.69, repressing that both stationary and mobile sources of emission can be considered as the main sources for benzene and toluene. Moreover, strong photochemical activity in Tehran was demonstrated by the high ratio of E/o-X. Meta-regression indicates that the concentration of BTEX has insignificantly (*p*-value > 0.05) increased over time. The BTEX compounds based on the target hazard quotient (THQ) were ordered as benzene > o-xylene > ethylbenzene > toluene. Percentile 95% of THQ due to benzene (4.973) and o-xylene (1.272) was higher than a value of 1. Percentile 95% excessive cancer risk (ECR) for benzene (1.25 × 10^6^) and ethylbenzene (1.11 × 10^6^) was higher than a value of 1.00 × 10^6^. The health risk assessment indicated that the population of Tehran are at considerable non-carcinogenic and carcinogenic risks.

## 1. Introduction

The air quality in urban areas depends on different factors such as atmospheric dispersion conditions, solar radiation, meteorological factors, geographical factors, deposition, and pollutant emissions [1]. In the last few decades, with increases in urbanization and developments in human life, the issue of air pollution has attracted considerable attention [2,3]. The primary sources of air pollution in urban regions can be summarized as natural and anthropogenic sources [4,5]. In this context, air pollutants such as volatile organic compounds (VOCs), sulfur oxides (SOx), ozone (O_3_), carbon oxides (COs), particulate matter (PM), nitrogen oxides (NOx), and radioactive pollutants are released from these sources [5,6]. 

The main sources of VOCs are anthropogenic and biogenic sources [7], including incomplete combustion in motor vehicles (fossil fuels), the petrochemical process, the fabrication of rubber and resin, solvents, and paint industries [8,9]. The presence of VOCs in a variety of forms such as toluene, benzene, ethylbenzene, and meta (m), para (p), and ortho(o) xylene in the indoor or outdoor air is important issue due to the consequence of non-carcinogenic risks (e.g., neurological impairment, allergy, nose and eye irritation, kidney and liver dysfunction, and heart disease) [10,11,12] and carcinogenic risks (e.g., lung cancer and leukemia) [13,14,15,16]. 

Among VOCs, benzene, as a hazardous compound with a relatively long lifetime, belongs to group 1; carcinogenic to humans [17,18]. Whilst the mutagenicity and carcinogenicity of toluene, ethylbenzene, and xylenes have not been proven [19,20], they are precursors of toxic radical in the atmosphere [21]. In this regard, in addition to direct adverse health effects of BTEX, they can be classified as the main precursors of the production of secondary pollutants by photochemical reactions such as proxy acetyl nitrate (PAN) and O_3,_ which can endanger human health [4,22,23,24,25,26,27].

Several investigations have been performed regarding measuring the concentration of ambient BTEX around the world and also to assess the quality and quantity of air pollutants and their effects on human health [24,28,29,30,31,32,33,34]. In this regard, useful information considering ratios used for determining photochemical activity in the atmosphere, as well as the sources of substances such as benzene/toluene (B/T) and ethylbenzene/m, p-xylene (E/X), were provided [8].

Although Tehran, with more than nine million permanent people and three million floating people, was designed for more than 750,000 motor vehicles, more than four million motor vehicles are moving in this metropolitan [35]. Besides, several factories are located in southern Tehran, with the emission of various pollutants into the ambient air [36]. Despite a high number of conducted studies regarding the concentration and numerous emission sources for BTEX in Tehran ambient air [37,38,39,40,41,42,43], no systematic review and meta-analysis study has been conducted to assess the related health risks. Therefore, for the first time, in the current study, the carcinogenesis and non-carcinogenesis risks of BTEX pollutants in Tehran will be assessed by using a systematic review and meta-analysis approach.

## 2. Material and Methods

### 2.1. Strategy of Search

The search strategy was performed to obtain all citations regarding the concentration of BTEX in the air of Tehran between 2005 to 2018. The systematic review was conducted based on the Cochrane method [44] using international databases including PubMed, Scopus, and Embase, and national databases including the Scientific Information Database (SID). The following keywords were used: (a) PubMed (Medline): ((((((((((benzene[Tit/Abs]) OR toluene[Tit/Abs]) OR ethylbenzene[Tit/Abs]) OR xylene[Tit/Abs]) OR BTEX[Tit/Abs]) OR volatile organic compound[Tit/Abs])) AND (((air pollution[Tit/Abs]) OR ambient air[Tit/Abs]) OR outdoor air[Tit/Abs])) OR air pollution[MeSH Terms]) AND Iran[Tit/Abs]) OR Iran[MeSH Terms]; (b) Scopus: ((keyword (benzene) or keyword (toluene) or keyword (ethylbenzene) or keyword (xylene) or keyword (BTEX) or keyword (volatile and organic and compound))) and ((keyword (air and pollution) or keyword (ambient and air) or keyword (outdoor and air))) and (keyword (Iran)); Embase: ‘benzene’:ab,ti OR ‘toluene’:ab,ti OR ‘ethylbenzene’:ab,ti OR ‘xylene’:ab,ti OR ‘btex’:ab,ti OR ‘volatile organic compound’:ab,ti AND ‘air pollution’/exp OR ‘air pollution’ OR ‘ambient air’:ab,ti OR ‘outdoor air’/exp OR ‘outdoor air’ AND ‘IRAN’:ab,ti. Thirteen years (1 January 2005 and 11 June 2018) was selected as the period of investigation.

### 2.2. Screening of Articles

The evaluation of initially retrieved articles was performed independently according to (1) title, (2) abstract, and (3) full-text of the articles [45,46]. According to the title and abstract, some articles that did not perform investigations on the concentration of BTEX in the air of Tehran were excluded.

The full text of the obtained papers was downloaded, after the abstract screening. Criteria for including articles were: (1) descriptive study on the contamination of BTEX; (2) full text available; (3) original studies; and (4) reporting of the concentration of BTEX in ambient air in Tehran. Disagreement among two of the authors was resolved by discussion; otherwise, a third author decided. A reference list of retrieved articles was also checked to obtain more articles. The required management of obtained references was carried out using EndNote X7^®^ (Thomson Reuters, Toronto, Canada) software [46]. 

### 2.3. Data Extraction and Definitions

The collected data from each article can be summarized as sampling date, type of monitoring station, number of monitoring stations, sample size, the concentration of BTEX, the method of detection, the limit of detection, and the limit of quantitation (Table 1). BTEX represents volatile chemicals including benzene, toluene, ethylbenzene, and xylene that are emitted from crude oil, natural gas, and petroleum deposits [47]. In this regard, because of the majority of studies performed on O-xylene, it was extracted from obtained articles. 

### 2.4. Meta-Analysis

While the heterogeneity was higher than 50%, the random effect model (REM) was used to estimate the pooled concentration of BTEX in ambient air [45,48].

The standard error (SE) of the concentration of BTEX was calculated using standard deviation and sample size (SE = SD/√n) [45]. According to the mean and standard error, the pooled concentration of BTEX was estimated [49,50]. All data were analyzed using STATA 14.0 statistical software (College Station, TX, USA). *p*-value < 0.05 was considered statistically significant.

### 2.5. Health Risk Assessment

#### 2.5.1. Non-Carcinogenic Risk

In the current study, according to part A and B of the risk assessment manual of EPA, the exposure dose to BTEX in ambient air was estimated [51]. Dose exposure via the inhalation [exposure concentration (EC)] pathway was calculated by Equation (1) [52,53].

(1) $$\;\mathrm{EC}=\frac{C\;\times \;\mathrm{ET}\;\times \;\mathrm{EF}\;\times \;\mathrm{ED}\;}{\;\mathrm{ATn}\;}$$

All parameters used in this equation are presented in Table 2.

The conversion of the concentration from ppb to µg/m^3^ for benzene, toluene, ethylbenzene, and o-xylene was performed using 3.243, 0.843, 19.45, and 4.33 convert coefficients, respectively [59].

To estimate the non-carcinogenic risk of BTEX in the ambient air, the target hazard quotient (THQ) was calculated using Equation (2) [51]:

(2) $$\;\mathrm{THQ}=\;\frac{\mathrm{EC}\;}{\mathrm{RfCi}\;\times 1000}$$

The total target hazard quotient (TTHQ) is equal to the sum of individual THQ [60,61,62,63,64,65,66]. The TTHQ of BTEX was calculated by Equation (3):

TTHQ = THQ_b_ + THQ_t_ + THQ_e_ + THQ_x_ (3)

When THQ and/or TTHQ is lower than or equal to a value of 1, the population are not at a significant non-carcinogenic risk [17].

#### 2.5.2. Carcinogenic Risk

The carcinogenic risk of benzene and ethylbenzene in adults and children was estimated using Equation (4):

(4)  ECR=(EC ×1000 ) ×IUR

The related parameters of Equations (1)–(4) are shown in Table 2.

When the ECR value of benzene and ethylbenzene is lower than 1.00 × 10^6^, between 1.00 × 10^6^ to 1.00 × 10^4^, and higher than 1.00 × 10^4^, the exposed population are at no considerable, threshold, and considerable cancer risk, respectively [54]. In the current study, the cut off point for endangering the population was a percentile of 95% (worse scenario) of THQ and ECR [65]. 

## 3. Results and Discussion

### 3.1. The Process of Select Papers

**Figure 1:**
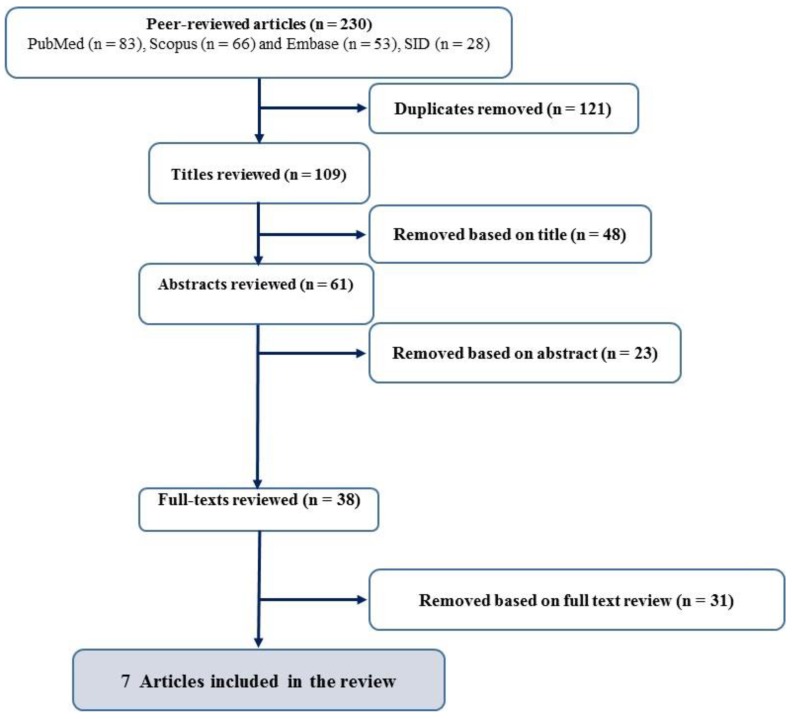
The selection process of articles and inclusion.

Among the 230 papers obtained published from 2005 to 2018 from all databases including PubMed (n = 83), Scopus (n = 66), Embased (n = 53), and SID (n = 28) in the identification step, 121 papers were excluded due to duplication. After the assessment of titles, 48 papers were regarded as unsuitable. The abstracts of 61 papers were checked, and 23 papers were excluded. Then, the full texts of the 38 papers were reviewed and finally, seven papers with 1678 samples were included in the current study (Figure 1).

### 3.2. Concentration of BTEX

**Figure 2:**
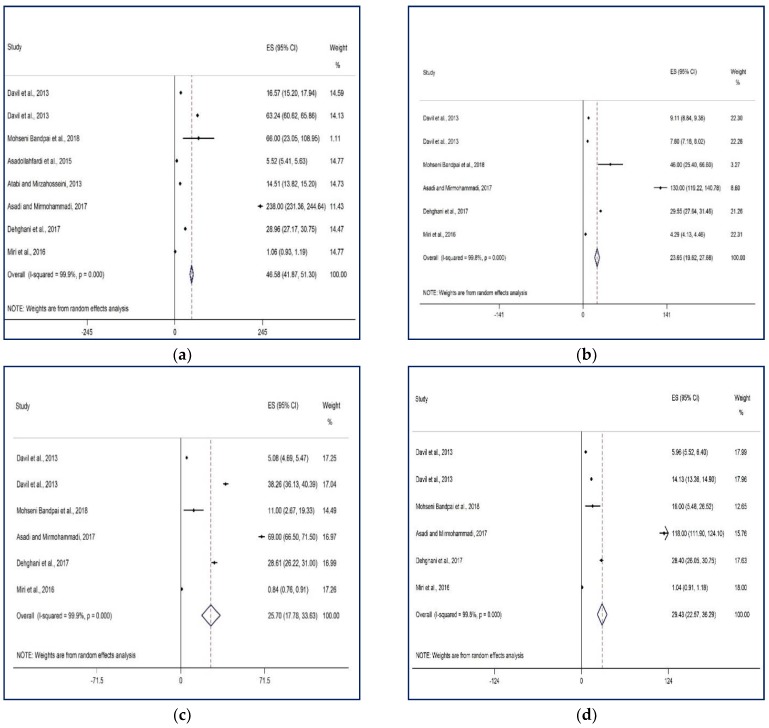
Forest plot of concentration (ppb) of benzene (**a**), toluene (**b**), ethylbenzene (**c**), and o-xylene (**d**) in the ambient air of Tehran. ES: effect size, CI: Confidence interval.

**Figure 3:**
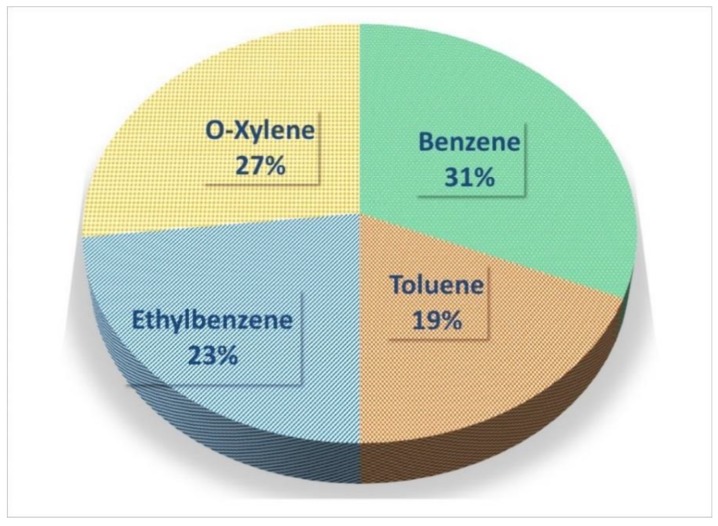
The percentage of BTEX in the ambient air of Tehran.

The pooled concentration (ppb) of benzene, toluene, ethylbenzene, and o-xylenes, is demonstrated in Figure 2a–d. The pooled concentration of benzene, toluene, ethylbenzene, and o-xylenes was 46.54 ppb (95% CI: 41.87–51.30 ppb), 23.65 ppb (95% CI: 19.62–27.68 ppb), 25.70 ppb (95% CI: 17.80–33.63 ppb), and 29.43 ppb (95% CI: 22.57–36.29 ppb), respectively. Also, the total BTEX concentration was measured as 125.13 ppb or 474.45 ± 29.93 µg/m^3^. The rank order based on their contribution in BTEX was benzene (46.54 ppb or 149.18 µg/m^3^: 31%) > o-xylene (29.43 ppb or 127.16 µg/m^3^: 27%) > ethylbenzene (25.70 ppb or 110.15 µg/m^3^: 23%) > toluene (23.65 ppb or 87.97 µg/m^3^: 19%) (Figure 3). 

A comparison of the concentration of BTEX in Tehran with other urban areas in the world is presented in Table 3. According to our findings, the pooled concentrations of benzene (149.18 µg/m^3^) and o-xylene (125.57 µg/m^3^) in Tehran were higher than those in other cities around the world (Table 3) [6,33,67,68,69,70,71,72,73,74] (Table 3).

The concentration of toluene in Tehran (87.97 µg/m^3^) was lower than that in Malaysia (Kuala Lumpur) (113.805 µg/m^3^) [73], but higher than other cities [6,33,67,68,69,70,71,72,74]. However, the concentration of ethylbenzene (110.12 µg/m^3^) in Tehran was lower than the reported value for Malaysia (Kuala Lumpur) (661.3 µg/m^3^) [73], but was lower than other cities (Table 3) [6,33,67,68,69,70,71,72,74].

The toluene levels in Italy (Bari) (4.76 ± 3.41 µg/m^3^), Canada (Sarnia) (2.88 µg/m^3^), Turkey (Kocaeli) (35.51 ± 39.55 µg/m^3^), China (Beijing) (2.21 ± 2.10 µg/m^3^), and Spain (Navarra) (13.26 µg/m^3^) were higher than other VOC compounds (Table 3) [6,67,68,69,70,71,72]; however, the concentrations of ethylbenzene in South Korea (Seoul) (80.75 µg/m^3^) and Malaysia (Kuala Lumpur) (661.3 µg/m^3^) were higher than other VOC compounds [33,73,74] (Table 3).

The concentration of BTEX in ambient air of Tehran was higher than many urban regions in the world (Table 3). The inversion phenomenon was mentioned as one of the leading causes of the high concentration of BTEX in Tehran [37,38,40,42]. It occurs in the cold seasons that cause ambient air pollutants such as VOCs to become trapped in the surface layer of the Earth, which can result in intensifying air pollution levels [37].

In addition to the inversion phenomenon, fossil fuel consumption of old vehicles, low-quality fuel, population congestion, non-standard streets, and highways, besides several factories in the south of Tehran such as iron and steel industries, are other reasons for the high level of BTEX in Tehran city [37,38,40,42].

The higher concentration of toluene and ethylbenzene in Malaysia (Kuala Lumpur) compared with Tehran is due to the higher evaporation of petrol vapors (gasoline evaporation) and emission of higher concentrations of toluene and ethylbenzene by motor vehicles [73].

### 3.3. The Ratio between BTEX Compounds

The ratios of benzene/toluene (B/T) and ethylbenzene/m, p-xylene (E/X) can be used to assess the photochemical activity in the atmosphere and sources [8,75]. The ratio between BTEX compounds is the main parameter for discovering the emission sources of BTEX in the outdoor air [76,77]. The calculated B/T ratio in the range of 0.23–0.66 shows that vehicles and traffic are the main emission sources of toluene and benzene in the ambient air of Tehran [8,75,78,79]. The B/T ratio lower than this range indicates that toluene and benzene mainly originated from stationary sources. Likewise, if the B/T ratio is higher than this range, stationary (factory) and mobile (Motorcycle and car) sources are the main sources of emission [8,75]. Ratios of benzene/toluene (B/T) and ethylbenzene/o-xylene (E/o-X) in Tehran and other areas are presented in Table 4. 

The B/T ratio in this study was 1.69, which was higher than 0.23–0.66, representing that the primary sources of benzene and toluene could be both stationary and mobile sources of emission. Similar to our study, the B/T ratio in ambient air in France (Orleans) and China (Beijing) was higher than the B/T ratio in this study (0.66) [24,33,70,85]. The B/T ratio in ambient air of China (Hong Kong), France (Paris), South Korea (Seoul), Turkey (Kocaeli), and Spain (Navarra) [25,32,70,71,80] was lower than 0.23, indicating that toluene and benzene mainly originated from stationary sources [8,75]. 

The ratio of E/X is a good indicator that indicates the degree of photochemical reactions [86,87]. A higher ratio of E/X than Spain (Navarra: 0.82), Turkey (Kocaeli: 0.78), and Italy (Bari: 0.71) shows that photochemical activity in the ambient air of Tehran is stronger than in Spain, Turkey, and Italy (Table 4) [88].

### 3.4. Health Risk Assessment

#### 3.4.1. Non-Carcinogenic Risk Assessment

Non-Carcinogenic Risk BTEX compounds are presented in Table 5. Percentile 95% of THQ of benzene, toluene, ethylbenzene, and o-xylene was determined as 5.342, 0.021, 0.142, and 1.522, respectively (Table 5). The rank order of BTEX compounds based on THQ was benzene > o-xylene > ethylbenzene > toluene. THQ of benzene was higher than other VOC compounds because the concentration of benzene was the highest (Table 5), and also its RfC_i_ was the lowest [55].

**Figure 4:**
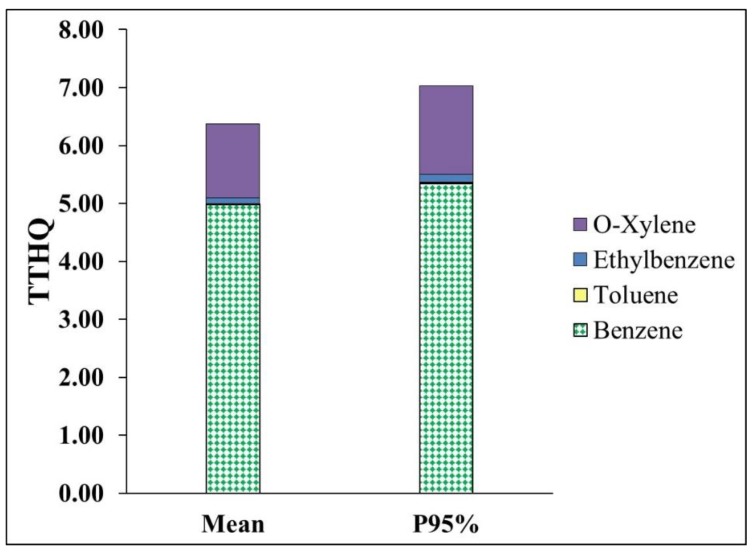
TTHQ of BTEX in ambient air due to inhalation exposed population.

Percentile 95% of THQ of benzene and o-xylene was higher than the value of 1. Also, TTHQ values based on mean and percentile 95% was 6.37 and 7.07, respectively, which were higher than a value of 1 (Figure 4). The health risk assessment shows that the residents of Tehran are at a considerable non-carcinogenic risk (THQ and TTHQ > 1 value). THQ values of benzene, toluene, ethylbenzene, and o-xylene in the China (Beijing) city were 3.2 × 10^2^, 3.37 × 10^4^, 3.19 × 10^4^, and 1.5 × 10^3^, respectively [70], which were lower than Tehran city. The lower concentration of BTEX in ambient air of China (Beijing) city (Table 3) was the main source of the lower non-carcinogenic risk when compared to Tehran city. 

#### 3.4.2. Carcinogenic Risk Assessment

The result of the carcinogenic risk assessment of benzene and ethylbenzene is presented in Table 6. Percentile 95% ECR of benzene and ethylbenzene was 1.25 × 10^6^ and 1.11 × 10^6^, respectively (Table 6). Also, percentile 95% ECR of benzene and ethylbenzene was higher than the value of 1.00 × 10^6^. In this context, the population of Tehran is at considerable carcinogenic risk. These outcomes of the health risk assessment show that strict monitoring needs to be performed to control the concentration of BTEX in ambient air in Tehran city and BTEX standards should be revised as soon as possible.

## 4. Conclusions

In the current study, the concentration of BTEX in Tehran ambient air was estimated based on a systematic review and meta-analysis approach and the non-carcinogenic and carcinogenic risks in the exposed population were estimated. The rank order of BTEX based on their concentration was benzene > o-xylene > ethylbenzene > toluene. The primary sources of benzene and toluene in ambient air of Tehran include both mobile and stationary sources of emission. Also, strong photochemical activities in the ambient air of Tehran were assumed. A health risk assessment based on the worse scenario (Percentile 95% THQ and ECR) indicated that the population of Tehran are at considerable non-carcinogenic and carcinogenic risks. Therefore, to reduce the health risks of BTEX, emission reduction plans should be implemented.

## Figures and Tables

**Table 1:** Main characteristics included in the study.

Sampling Date		Type of Monitoring Station	Monitoring Station Number	Sample Size	Concentration								Method of Detection	Ref
					Benzene		Toluene		Ethyl Benzene		O-Xylene			
Start Time	End Time				Average	SD	Average	SD	Average	SD	Average	SD		
23-November	22-December-2007	Urban	1	70	16.57	5.86	9.11	1.16	5.08	1.67	5.96	1.89	VOC71M-PID	[37]
10-December	9-January-2008	Urban	1	70	63.24	11.19	7.6	1.78	38.26	9.08	14.13	3.29	VOC71M-PID	[37]
1-December	30-December-2015	Urban	1	20	66	98	46	47	11	19	16	24	GC/FID	[38]
July	September-2010	Urban	14	948	5.52	NM ^1^	NM	NM	NM	NM	NM	NM	NM	[39]
5-April-2010	25-March-2011	Traffic	16	80	14.51	3.17	NM	NM	NM	NM	NM	NM	VOC71M-PID	[40]
February	July-2015	Urban	46	360	238	NM	130	62.04	69	NM	118	NM	GC/FID	[41]
November-2014	March-2015	Traffic	1	100	28.96	9.12	29.55	9.73	28.61	12.2	28.4	12.01	GC/FID	[42]
March-2012	March-2013	Urban	7	30	1.056	NM	4.291	NM	0.837	NM	1.044	NM	GC/FID	[43]

^1^ Not Mentioned.

**Table 2:** Parameters included in the health risk assessment due to the exposure inhalation pathway.

Parameter	Define	Unit	Value	Reference
EC	Chronic and sub-chronic exposure concentration	µg m^−3^	─	[54]
C	Concentration	mg m^−^^3^	─	
THQ	Target Hazard Quotient	Unitless	─	[54]
TTHQ	Total target Hazard Quotient	Unitless	─	
IUR_Bap_	Inhalation unit risk	((mg m^−^^3^)^−1^)	Benzene: 7.8 × 10^6^ Ethylbenzene: 2.5 × 10^6^	[55]
ECR	Excessive cancer risk	Unitless	Benzene: 0.030	[55]
RfCi	Inhalation reference concentrations	mg m^−3^	Toluene: 5.000 Ethylbenzene: 1.000 O-Xylene: 0.100	[55]
EF	Exposure frequency	day year^−1^	180	[56]
ED	Exposure duration	year	Adults: 24	[57]
ET	Exposure time	hour day^−1^	24	[52]
ATn	Averaging time	days	ATn = Non-carcinogens: ED × EF days	[58]
IUR	Inhalation unit risk	(µg m^−^^3^)^−^^1^	Benzene = 7.80 × 10^6^ Ethylbenzene = 2.50 × 10^6^	[55]
1000	Convert factor mg to µg	─	─	

**Table 3:** A comparison of the concentration of BTEX in ambient air of Tehran with other regions in the world (µg/m^3^).

City/Country	Sample Size	Monitoring Periods	Benzene	Toluene	Ethylbenzene	O-Xylene	Method	Type of Source	References
Bari/Italy	NM ^1^	April, September and October 2008	2.29 ± 1.59	4.76 ± 3.41	0.92 ± 0.66	1.3 ± 0.94	GC/MS	Urban	[67]
18 areas/Canada	NM	September 2009 and December 2011	0.58	1.55	0.24	0.24	GC/MS	Urban	[68]
Aliaga/Western Turkey	13	2005 and 2007	0.68 ± 0.68	1.6 ± 1.1	0.25 ± 0.17	0.16 ± 0.13	GC/FID	Urban	[69]
Kocaeli/Turkey	49	July 2006	2.26 ± 3.20	35.51 ± 39.55	9.72 ± 9.20	12.46 ± 12.46	GC/FID	Urban	[6]
Beijing/China	41	26 February and 7 March 2013	1.73 ± 1.68	2.21 ± 2.10	0.38 ± 0.38	0.19 ± 0.17	GC/FID	Urban	[70]
Orleans/France	56	Winter 2011	0.95	0.27	0.95	0.14	(TD–GC–MSD)	Semi-urban	[33]
Navarra/Spain	932	June 2006 to June 2007	2.84	13.26	2.15	2.63	GC/MS	Urban	[71]
Sarnia/Canada	37	2004–2005	0.93	2.54	0.46	0.49	GC/MS	Urban	[72]
Windsor/Canada	42	2004–2005	0.76	2.88	0.44	0.45	GC/MS	Urban	[72]
Kuala Lumpur/Malaysia	28	December 2013 and January 2014	58.374	113.805	661.3	NM	GC/MS	Urban	[73]
Seoul/South Korea	8003	2004	2.829	32.76	80.75	NM	GC/FID	Urban	[74]
**Present study**	**1678**	**2007–2015**	**149.18**	**87.97**	**110.12**	**127.14**			

^1^ Not Mentioned.

**Table 4:** Comparison of benzene/toluene (B/T) and ethylbenzene/o-xylene (E/o-X) concentration ratio in Tehran and other areas.

Area Study	B/T	E/O-X	References
Guangzhou/China	0.35		[28]
Hong Kong/China	0.13		[80]
Helsinki/Finland	0.36		[81]
Munich/Germany	0.53		[82]
Louis/India	0.93		[83]
Paris/France	0.15		[32]
London/English	0.65		[84]
Seoul/South Korea	0.13		[25]
Beijing/China	0.71		[85]
Beijing/China	0.87		[24]
Bari/Italy	0.48	0.71	[67]
18 areas/Canada	0.37	1.00	[68]
Aliaga/Western Turkey	0.43	1.56	[69]
Kocaeli/Turkey	0.06	0.78	[6]
Beijing/China	0.78	2.00	[70]
Orleans/France	3.49	6.50	[33]
Navarra/Spain	0.21	0.82	[71]
Sarnia/Canada	0.37	0.94	[72]
Windsor/Canada	0.26	0.98	[72]
Kuala Lumpur/Malaysia	0.51		[73]
Tehran/Iran	1.69	0.86	Present study

**Table 5:** Non-carcinogenic risk due to the inhalation of benzene, toluene, ethylbenzene, and o-xylene.

VOCs Compounds	C (Mean)	C (P95%)	EC (Mean)	EC (P95%)	RfCi	THQ (Mean)	THQ (P95%)
	µg/m^−3^		µg/m^−3^		mg/m^3^		
Benzene	149.178	160.27	0.149	0.160	0.03	4.973	5.342
Toluene	87.970	107	0.088	0.107	5.00	0.018	0.021
Ethylbenzene	110.150	142	0.110	0.142	1.00	0.110	0.142
O-Xylene	127.160	152.2	0.127	0.152	0.10	1.272	1.522

**Table 6:** Carcinogenic risk due to the inhalation of benzene and ethylbenzene.

VOCs Compounds	EC (Mean)	EC (P95%)	IUR	ECR (Mean)	ECR (P95%)
Benzene	0.149	0.160	7.80 × 10^6^	1.16 × 10^6^	1.25 × 10^6^
Ethylbenzene	0.110	0.142	2.50 × 10^6^	8.59 × 10^7^	1.11 × 10^6^
